# Determinants of hospital readmissions in older people with dementia: a narrative review

**DOI:** 10.1186/s12877-024-04905-6

**Published:** 2024-04-12

**Authors:** Bria Browne, Khalid Ali, Elizabeth Ford, Naji Tabet

**Affiliations:** 1 Centre for Dementia Studies, Brighton and Sussex Medical School, The University of Sussex Brighton, Brighton, UK; 2 Department of Medicine, Brighton and Sussex Medical School, Brighton, UK; 3 Department of Elderly Care and Stroke Medicine, University Hospitals Sussex NHS Trust, Brighton, UK; 4 Department of Primary Care and Public Health, Brighton and Sussex Medical School, Brighton, UK

**Keywords:** Dementia, Hospital readmissions, Hospitalisations, Determinants, Narrative review, Older adults, Psychosocial

## Abstract

**Introduction:**

Over 50% of hospitalised older people with dementia have multimorbidity, and are at an increased risk of hospital readmissions within 30 days of their discharge. Between 20-40% of these readmissions may be preventable. Current research focuses on the physical causes of hospital readmissions. However, older people with dementia have additional psychosocial factors that are likely to increase their risk of readmissions. This narrative review aimed to identify psychosocial determinants of hospital readmissions, within the context of known physical factors.

**Methods:**

Electronic databases MEDLINE, EMBASE, CINAHL and PsychInfo were searched from inception until July 2022 and followed up in February 2024. Quantitative and qualitative studies in English including adults aged 65 years and over with dementia, their care workers and informal carers were considered if they investigated hospital readmissions. An inductive approach was adopted to map the determinants of readmissions. Identified themes were described as narrative categories.

**Results:**

Seventeen studies including 7,194,878 participants met our inclusion criteria from a total of 6369 articles. Sixteen quantitative studies included observational cohort and randomised controlled trial designs, and one study was qualitative. Ten studies were based in the USA, and one study each from Taiwan, Australia, Canada, Sweden, Japan, Denmark, and The Netherlands. Large hospital and insurance records provided data on over 2 million patients in one American study. Physical determinants included reduced mobility and accumulation of long-term conditions. Psychosocial determinants included inadequate hospital discharge planning, limited interdisciplinary collaboration, socioeconomic inequalities among ethnic minorities, and behavioural and psychological symptoms. Other important psychosocial factors such as loneliness, poverty and mental well-being, were not included in the studies.

**Conclusion:**

Poorly defined roles and responsibilities of health and social care professionals and poor communication during care transitions, increase the risk of readmission in older people with dementia. These identified psychosocial determinants are likely to significantly contribute to readmissions. However, future research should focus on the understanding of the interaction between a host of psychosocial and physical determinants, and multidisciplinary interventions across care settings to reduce hospital readmissions.

**Supplementary Information:**

The online version contains supplementary material available at 10.1186/s12877-024-04905-6.

## Background

Dementia is a known public health priority due to the increasing amount of people living with the condition worldwide and the subsequent demands placed on health and social care systems, the economy and society [1]. The World Alzheimer’s Report estimated that over 46 million people were living with dementia worldwide in 2015, and this figure is estimated to increase to 131.5 million by 2050 [2]. In the United Kingdom, there are currently over 910,000 people living with dementia (PLWD), and this number is projected to increase to over 2 million by 2051, with dementia care costs estimated to have an overall economic impact of £26 billion per annum [3, 4]. Dementia has a complex relationship with long-term conditions, with adverse impacts on older PLWD [5]. This health and social care challenge is demonstrated by UK Hospital Episode Statistics (HES) data in research by Age UK and the National Institute for Health and Care Research (NIHR), where 53% of hospitalised PLWD have three or more long-term conditions [6]. Additionally, the negative impact of psychosocial factors such as social isolation, poor dementia-friendly environments and behavioural and psychological symptoms of dementia (BPSD), may also contribute towards increased hospital readmissions in PLWD. People with dementia who live in areas of social and economic deprivation or live with depression or anxiety, are at a greater risk of avoidable healthcare outcomes including hospital readmissions [7, 8]. Recurrent admissions could lead to faster deterioration, poor quality of life and increased mortality risk for PLWD [9].

Analysis from the UK HES data commissioned by Alzheimer’s Research UK, found that the number of PLWD aged 65 and over being admitted into hospitals increased by 93% from 210,000 admissions in 2010/11 to 405,000 in 2017/18 [10]. These hospitalisations could be related to the increased severity of long-term conditions, care needs at the point of discharge, and inadequate resources in post-discharge care [11]. These figures escalate pressure on healthcare services in caring for PLWD. Additionally, analysis of NHS data by the Alzheimer’s Society found that older people with dementia remain in hospital for up to seven times longer than their age-matched groups without dementia [12].

Hospital readmission in older adults is recognised as an unplanned return admission to an acute care hospital, within 30 days of their previous admission [13, 14]. International research studies indicate that PLWD are more likely to be readmitted to hospital within 30 days of their index admission, where 20–40% of these readmissions are avoidable [11, 15–17]. Previous literature reviews focused on the physical determinants of hospital readmissions, such as multimorbidity [16, 18, 19]. However, PLWD also require support with their psychosocial needs in addition to their physical needs. Throughout the dementia trajectory, biomedical deterioration such as frailty and multimorbidity progress in conjunction with psychosocial deterioration, including depression and poor social support [20]. Cohen-Mansfield [21] proposed one of the first biopsychosocial models of dementia, where dementia manifests from predisposing factors, life-long events and current biological, psychological and environmental factors. It was suggested that these factors affect the trajectory of dementia through cognitive, behavioural, self-maintenance and affective functioning. For example, pain is a common physical condition with psychosocial impact on cognitive, behavioural and affective functioning for PLWD [22]. Spector and Orrell [23] adapted the biopsychosocial model of dementia, where they proposed the impact of biopsychosocial factors varying along the dementia trajectory from normal ageing to the end of life. Hence, the same biopsychosocial factors may have different effects depending on the cognitive status of PLWD [23]. This is evident as hospital readmissions have been found to increase towards the end of life for PLWD, with various factors including poor community support, pain and multimorbidity [24]. Therefore, focusing on a few factors for hospital readmissions is problematic, as the dementia trajectory and health outcomes for PLWD are based on the interaction of a myriad of biomedical and psychosocial determinants [25]. Understanding the psychosocial determinants of hospital readmissions in PLWD may provide individual benefits as well as service implications in reducing this problem.

This narrative review aims to identify the psychosocial determinants of hospital readmissions in older PLWD, within the context of the known physical determinants.

## Methods

A review was conducted to develop a holistic understanding of the determinants of hospital readmissions among older PLWD. The methodology of this review followed the framework outlined by Arksey and O’Malley [26]. The reporting followed the Preferred Reporting Items for Systematic Reviews and Meta-Analyses extension for Scoping Reviews (PRISMA-ScR), to provide a scientific approach to this review [27]. A protocol was registered with the Zenodo repository (DOI number: 10.5281/zenodo.10044172).

### Step one: identifying the research question

This narrative review was guided by the following research question:

- What are the psychosocial determinants for acute hospital readmissions for older people with dementia, within 30 days of an index admission?

### Step two: identifying relevant studies

A defined search strategy was used for the electronic databases MEDLINE, EMBASE, CINAHL and PsychInfo, which were searched from inception to July 2022. A follow-up search of the electronic databases was carried out in February 2024, to retrieve the latest publications. Search terms for dementia and hospital readmissions were used in combination with truncation and Boolean operators, including AND and OR, to yield results. The search terms used were followed by the dementia literature search strategies guidance, by the National Institute for Health and Care Excellence (NICE) [28]. To ensure that further relevant articles were identified within the search, the citations of all included studies were searched and screened for inclusion. An initial pilot search was conducted in June 2022, to refine the search strategy and ensure that the search terms produced relevant results. This technique was used to ensure that the search was specific enough to answer the research question, and broad enough to obtain the relevant literature, including non-psychological determinants for readmissions. No restrictions were placed on the publication year. The full search applied to the MEDLINE database is outlined in supplementary data 1.

### Step three: study selection

Publications were included in the review if they involved:

- Adults aged 65 years and over living with a dementia diagnosis.
- Health and social care workers who work in dementia care, within hospital and community settings.
- Family carers of people with dementia who have experienced hospital readmissions.
- All empirical studies including quantitative and qualitative research.

Publications were excluded for the following reasons:

- Hospital readmissions that do not include people with dementia.
- Adults aged under 65 years living with dementia.
- Conference abstracts, theses, editorials and opinion pieces.
- Articles not in English language.

All publications retrieved from the search strategy were imported into Endnote 20 software to remove duplications and manage references. All titles and abstracts were screened against the eligibility criteria by two independent reviewers (BB and NT). Eligible titles and abstracts then had full-text screening against the eligibility criteria, by the two independent reviewers (BB and NT). Where there was any disagreement among the reviewers, one reviewer (BB) re-read the full-text publication against the eligibility criteria and a consensus was reached by the reviewers.

### Step four: charting the data

A data extraction form was developed on Microsoft Excel and pilot-tested on five publications by random selection, to ensure that the relevant data were extracted from the publications. Details on the data extraction form for included publications included study title, author and year, country, study design and aim, research setting, data source, study period, sample characteristics, readmission measure and rate, and reasons for hospital readmissions (Table 1). One reviewer (BB) completed the data extraction for all included publications, which was reviewed and discussed with a second reviewer (NT), for overall agreement of data extraction.

### Step five: collating, summarising, and reporting the results

The data were cross-tabulated and compiled on a spreadsheet in Microsoft Excel for narrative synthesis to be conducted. As the measurements of the determinants of hospital readmissions in older PLWD were variable such as all-cause 30 and 180-day readmission rates, frequencies of rehospitalisations and successful discharge-to-community rates, methods of statistical pooling data were not feasible.

A narrative synthesis approach was used to allow the integration of quantitative and qualitative data, to identify patterns related to hospital readmissions in older PLWD. Two elements of narrative synthesis outlined by Popay et al. [29] were adopted. The two elements used were ‘*developing a preliminary synthesis’* and ‘*exploring relationships in the data’*. The elements ‘*developing a theory of how and why interventions work’* and ‘*assessing the robustness of the synthesis’* were not used, as this narrative review was exploratory in nature and assessing the quality of studies was not required to map the relevant evidence available. The summarising of data was completed by one reviewer (BB) and discussed further with a second reviewer (NT), to gain agreement on the developed narrative categories and to report the findings.

## Results

### Summary of results

The search strategy yielded 4757 articles. After duplicates were removed and the inclusion of five additional studies from citation searching, 4736 titles and abstracts were screened. Thirty articles underwent full-text screening, and fourteen studies were excluded because they did not meet the inclusion criteria. Reasons for exclusion included the age of people with dementia being as young as 30 years old [30], a study based on hospital discharge issues with no relation to hospital readmissions [31], studies investigating initial hospitalisations instead of recurrent admissions [7, 24, 32–37], and the research being published as PhD theses [38, 39]. The full-text versions of two articles could not be located [40, 41]. After the follow-up search, 1633 titles and abstracts were screened. Three articles underwent full-text screening. Among this subgroup, two studies did not meet the inclusion criteria as they included PLWD aged under 65 years old [42, 43].

**Fig. 1:**
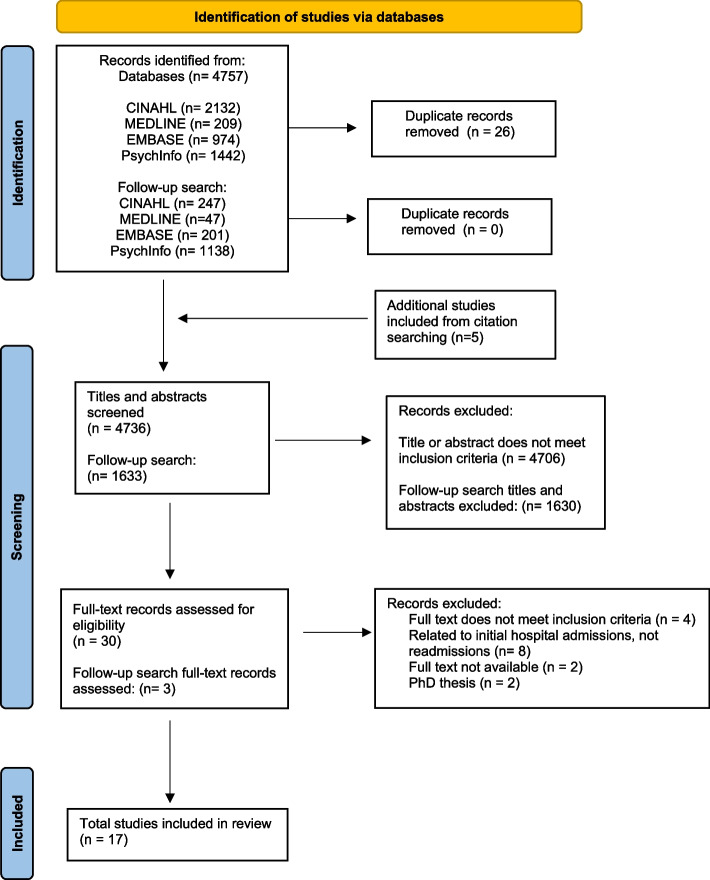
PRISMA flowchart of the study selection process

In total, 17 articles were included in this review. A flow chart of the screened studies is shown in Fig. 1.

### Study characteristics

Table 1 provides the characteristics of all 17 studies included in this narrative review, namely 15 cohort studies [16–19, 44–54], one qualitative study [55] and one randomised controlled trial [56]. A total of 7,194,878 participants were included in this review. The high number of participants is explained by the fact that large hospital and insurance records were used in 15 out of 17 studies [16–19, 44–47, 49–53, 56, 57], with three studies each having over 1.5 million participants [18, 51, 57]. The studies were undertaken in eight countries: one in Taiwan [44], ten in the USA [45–51, 53–55], one in Australia [52], and one each in Canada [17], Sweden [56], Japan [18], the Netherlands [19], and Denmark [16].

**Table 1:** Study characteristics

Author (year)^a^	Study title	Study design, setting, time period, country	Study aim	Data source	Sample characteristics	Readmission measure & readmission rate	Reasons for hospital readmissions & other findings
Cummings (1999) [45]	Adequacy of discharge plans & re-hospitalisation among hospitalised dementia patients	Prospective cohort, hospital, 1996–1997, USA	To measure the adequacy of overall discharge plans developed for older adults with dementia	Hospital medical records	131 older inpatients with moderate-severe dementia. Social workers (SW) developed discharge plan Discharge plans rated by primary care providers 1 month post discharge	Measure: 30-day hospital readmission. Readmission rate: 18.3%	35% discharge plans rated less than adequate or very inadequate. Logistic regression of factors predicting readmission were SW adequacy ratings (*p* < 0.01), patient gender (*p* < 0.01), caregiver support (*p* < 0.05).
Rudolph et al. (2010) [49]	Hospitalisation in community-dwelling persons with Alzheimer’s Disease (AD): Frequency & causes	Longitudinal study, hospital, 1991–2006, USA	To clinically identify patients with AD at high risk for hospitalisation based on baseline risk factors	Hospital medical records	827 patients with AD aged 65 + years	Measure: First hospitalisation in study period. Rate: 66% hospitalised at least once, 47% hospitalised two or three times during study period	Leading reasons for hospitalisation include: syncope/falls (26%), ischaemic heart disease (17%), gastrointestinal disease (9%), pneumonia (6%) and delirium (5%). Significant independent risk factors for hospitalisation were higher comorbidity (HR 1.87), previous acute hospitalisation (HR 1.65), older age (HR 1.51), male sex (HR 1.27) shorter duration of dementia symptoms (HR 1.26).
Callahan et al. (2012) [50]	Transitions in care for older adults with and without dementia	Prospective cohort, transitional care, 2001–2008, USA	To gain a complex understanding of the frequency and type of transitions in care of older adults with and without dementia	Medical records	4,197 primary care older adult patients (1,523 with dementia)	Measure: 30-day rehospitalisation after index admission. Rate: 23% for older adults with dementia. 28% for older adults with dementia with 30-day or shorter rehospitalisation.	Of the 30-day re-hospitalisations, 17% were discharges from index hospitalisations to home with healthcare services, 38% were discharges to home without home healthcare services, and 45% were discharges to a nursing facility.
Daiello et al. (2014) [53]	Association of dementia with early re-hospitalisation among Medicare beneficiaries	Retrospective cohort, hospital, Jan-Dec 2009, USA	To investigate the risk of rehospitalisation among Medicare beneficiaries with and without dementia.	Medicare insurance claims records	16,244 Medicare beneficiaries	Measure: 30-day hospital readmission in a 12-month period Rate: 17.8% vs. 14.5% (dementia vs. no dementia)	Dementia diagnosis was a predictor of 30-day readmission (unadjusted OR 1.28). Association persisted after adjustment for age, gender, number of long-term conditions, length of first hospital stay, number of admissions in previous year (OR 1.18). Prescriptions for antipsychotic medication that were recorded at least twice in the study period occurred more frequently in PLWD with 30-day hospital readmissions (12.7% readmission vs. 9.2% no readmission *p* < 0.001).
Chang et al. (2015) [44]	The impact admission etiology on recurrent or frequent admission	Prospective cohort, hospital, 2007–2014, Taiwan	To explore the roles of dementia subtypes, cerebrovascular risk factors, systemic diseases and the etiology for admission in predicting recurrent and prolonged hospitalisation	Hospital medical records	203 patients aged over 65 years with Alzheimer’s disease, vascular dementia or Parkinsonism-related dementia diagnosis. Clinical Dementia Rating score of 1–2 (mild-moderate)	Length of admission ≧ 14 days per hospitalisation and admission frequency ≧ 4 times in 4 years Readmission rate: 8.9%	Coronary artery disease associated with frequent admission (*p* = 0.023). For admission etiology, pneumonia, UTI and falls-related fracture highly associated with frequent admission (all < 0.0001). Admission etiologies have higher clinical weighting than dementia subtype and co-existing medical conditions, to predict recurrent admission and prolonged hospital stay.
Tropea et al. (2016) [52]	Poorer outcomes and greater healthcare costs for hospitalised older people with dementia and delirium	Retrospective cohort, hospital, 2006–2012, Australia	To compare healthcare utilisation outcomes among older hospitalised patients with and without cognitive impairment (CI), and to compare the costs associated with these outcomes.	Hospital medical records	50,261 Hospital patients with cognitive impairment (defined by ICD-10 diagnoses codes for dementia and delirium)	Measure: 28-day readmission rate Rate: Initially no significant difference of 2, 7 or 28 day readmissions between CI and non-CI patients. However, when discharged back to usual place of residence/home, the odds of 2, 7 & 28 day readmission increased for CI patients (OR 1.32, 1.22, 1.27)	The total cost of index admissions and 28-day readmissions involving CI patients was 47% higher than the cost of episodes involving non-CI patients. Increased rate of readmission for CI patients when discharged back home may indicate inappropriate discharge and further bed-based/transitional care/rehabilitation was required, or inadequate support services in place for successful transition back home.
Gilmore-Bykovskyi et al. (2017) [55]	Transitions From Hospitals to Skilled Nursing Facilities (SNF) for Persons With Dementia: A Challenging Convergence of Patient and System-Level Needs	Qualitative interviews, nursing home, 2015, USA	To examine SNF nurses’ perspectives regarding experiences and needs of people with dementia during hospital to SNF transitions & to identify factors related to the quality of these transitions	SNF nurses	51 SNF nurses	N/A	Under-communication was perceived as resulting in inappropriate placement, risk for rehospitalisation & patient harm. Hospital time pressures to discharge quickly, differences in the emphasis of medical & social/behavioural needs between hospital & SNF settings, differing views on transparency about behavioural symptoms & what defines a successful transition between hospital and SNFs, were areas of conflict described from nurses between SNF and hospitals.
Gustafsson et al. (2017) [56]	Pharmacist participation in hospital ward teams and hospital readmission rates among PWD	RCT, hospital, 2012–2014, Sweden	To assess whether comprehensive medication reviews conducted by clinical pharmacists could reduce the rate of drug-related admissions among people with dementia	Medical records	460 patients aged 65 + with dementia or cognitive impairment	Measure: 30 and 180-day readmission 30-day readmission rate: 5% intervention group, 11% control group (*p* = 0.03) 180-day readmission rate: 11% intervention, 20% control (*p* = 0.02) [sub-grp analysis of patients without heart failure]	Pharmacist participation did not significantly reduce 180-day readmissions in patients with heart failure (HF) (maybe as HF is a severe condition and can have many exacerbations). Reasons for reduced 180-day and 30-day readmissions in patients without HF may be due to close collaboration between pharmacist and ward medical team, as the pharmacist was already working on the ward and known to the team before the study.
Lin et al. (2017) [51]	Hospitalisations for ambulatory care sensitive conditions and unplanned readmissions among Medicare beneficiaries with Alzheimer’s disease	Retrospective cohort, hospital, 2013, USA	To examine the frequency and costs of potentially avoidable hospitalisations and unplanned 30-day readmissions in the entire Medicare fee-for-service population with dementia	Hospital insurance records	2,749,172 adults aged 65 + years with dementia	Measure: 30-day hospital readmission. Rate: 18% Of this amount, 73% had one all-cause 30-day admission, whereas 18% were readmitted twice and 9% were readmitted three or more times	Readmissions varied by diagnosis of index admission: 22% for heart failure, 21% for COPD, 19% for acute myocardial infarction, 18% for coronary artery bypass graft, 15% for pneumonia, 12% stroke, 9% hip/knee replacement. In total, 410,000 dementia patients had 567,000 ambulatory care sensitive condition (ACSC) hospitalisations or unplanned readmissions in 2013, costing Medicare $374 million/year. Such hospitalisations may be indicative of access barriers, problems in continuity of care, inefficient resource use, and poor patient outcomes.
Sakata et al. (2018) [18]	Dementia and risk of 30-day readmissions in older adults after discharge from acute care hospitals	Retrospective cohort, hospital, 2014–2015, Japan	To assess the association between dementia and risk of hospital readmission, accounting for primary diagnosis as a possible effect modifier	Diagnosis procedure combination database	1,834,378 Adults over 65 with and without dementia with 2 or more hospital admissions within study period	Measure: 30-day unplanned hospital readmission. Readmission rate: 8.3% (dementia) vs. 4.1% (control)	Significant associations between dementia and hospital readmission among 17/30 most common diagnostic categories including pneumonia and heart failure. Hip fracture was associated with greater readmission risk for people with dementia (11.5% vs. 7.9%). Greater readmission risk could be due to limited ability to follow post-discharge directions which may lead to poor health outcomes and readmission.
Van de Vorst et al. (2018) [19]	Increased mortality and hospital readmission risk in patients with dementia & a history of cardiovascular disease (CVD)	Prospective cohort, day clinic and hospital, 2000–2010, Netherlands	To evaluate the impact of CVD on mortality & hospital readmission risk in hospitalised dementia patients	Hospital database records	59,194 patients with dementia. 36.9% with CVD history	Measure: all-cause 1 year readmission Readmission rate: Day clinic patients with CVD = 49.7%, no CVD = 40.6% Hospital patients with CVD = 37.3%, no CVD = 28.1%	The presence of CVD increases the risk of hospital readmission in both day clinic and hospitalised patients with dementia. Day clinic HR = 1.26 Hospital HR = 1.34
Knox et al. (2020) [47]	Function & caregiver support associated with readmissions during home health for individuals with dementia	Retrospective cohort, home care, 2013–2015, USA	To determine the association between mobility, self-care, cognition and caregiver support, and 30-day potentially preventable readmissions (PPR) for people with dementia	Medical records	118,171 individuals with dementia	Measure: 30-day potentially preventable readmission. 30-day PPR rate: 7.6%	People with dementia who were most dependent on mobility (OR 1.59), and self-care (OR 1.73) had higher odds for PPR. The largest difference in 30-day PPR rates were according to the number of acute hospital stays in the prior year with 4 + stays having a 30-day PPR rate of 25.2% compared to 2.5% with patients with no other acute stays in the previous year.
Godard-Sebillotte et al. (2021) [17]	Primary care continuity & potentially avoidable hospitalisations in people with dementia	Retrospective cohort, primary care, 2014–2015, Canada	To estimate the association between high primary care continuity & potentially avoidable hospitalisation in community-dwelling people with dementia. High primary care defined as having had every primary a visit with the same physician.	Healthcare data	22,060 Community-dwelling people with dementia aged 65+, not awaiting admission to a long-term care facility	Measure: 30-day hospital readmission Readmission rate: rr = 0.81 (*p* < 0.001)	Reasons for reduced hospitalisation: Primary care may improve management of chronic conditions & detection & treatment of acute exacerbations; increase the ability to identify & communicate acute symptoms between people with dementia and physicians.
Graversen et al. (2021) [16]	Dementia and the risk of short-term readmission and mortality after a pneumonia admission	Retrospective cohort, hospital, 2000–2016, Denmark	To investigate 30-day mortality and readmission after hospital discharge for pneumonia in people with vs. without dementia	Hospital medical records	25,948 people with dementia aged 65–99 years	Measure: 30-day hospital readmission Readmission rate: 21.9% in people with dementia (14.9% in those without dementia)	In people with dementia, the highest readmission risks were found in the first week post-discharge. Could reflect in-hospital factors i.e. illness burden inpatient care process factors, poor discharge planning.
Knox et al. (2021) [46]	Mobility and self-care are associated with discharge to community after Home Health for people with dementia	Retrospective cohort, home care, 2016–2017, USA	To determine the relationship between dementia, mobility, self-care tasks, caregiver support and medication management with successful discharge to community (DTC) after home health.	Medical records	790,439 Medicare beneficiaries (18% with dementia)	Measure: Successful DTC (discharged to community without experiencing readmission or death within 30 days of discharge). Successful DTC rate: Dementia = 71%. No dementia = 81%.	People with dementia had significantly lower odds of successful DTC compared to people without dementia (rr = 0.947). The primary reason for unsuccessful DTC in ADRD was readmission during home health care, followed by unplanned readmission after discharge from home health care and mortality. Living alone and inadequate care was shown to increase the likelihood of readmissions i.e. those with dementia who were dependent on caregiver assistance and unable to manage their own medication had a lower risk of successful DTC.
Tannenbaum et al. (2022) [54]	Hospital practices and clinical outcomes associated with behavioural symptoms in persons with dementia	Retrospective cohort, hospital, January-December 2019, USA	To identify clinical practices and outcomes associated with behavioural symptoms in hospitalised PLWD.	Electronic hospital records	8,876 hospitalised people with dementia aged 65 years and over	Measure: 30-day hospital readmission Readmission rate: 14% (*n* = 1305)	40.6% (*n* = 3606) of hospitalised PLWD had behavioural symptoms. PLWD with behavioural symptoms were more likely to be male (40.3% vs. 36.9%, *p* = 0.001), and White (62.7% vs. 58.3%, *p* < 0.001), and more likely to come from a care facility (26.6% vs. 23.7%, *p* < 0.05).
Gilmore-Bykovskyi et al. (2023) [57]	Disparities in 30-day readmission rates among Medicare enrolees with dementia	Retrospective cohort, hospital, 2014, USA	To examine the association between race and 30-day readmissions in Medicare beneficiaries of Black and non-Hispanic White people with dementia	Hospital insurance records	1,523,142 hospital stays. 86% non-Hispanic White, 14% Black	Measure: all-cause 30-day readmission Readmission rate among Black beneficiaries: 37% unadjusted, 16% adjusted	Black beneficiaries more likely to live in disadvantaged, urban neighbourhoods, and had twice as much disability compared to non-Hispanic Whites. 50% of observed risk of readmissions in Black people is determined by unmeasured exposures of racial differences.

^a^Studies sorted by year of publication, then by surname of first author

Table 2 summarises the mapping of included studies according to the main categories and sub-categories of the determinants of hospital readmissions in older PLWD. Regarding the psychosocial determinants, the majority of studies were included in the category *inadequate discharge planning* (*n* = 5), followed by *interdisciplinary collaboration* (*n* = 4), and *ethnic differences in dementia* (*n* = 1). The category *behavioural and psychological symptoms* was recognised to connect both psychosocial and physical determinants simultaneously (*n* = 2). Categories within the physical determinants included *long-term conditions* (*n* = 5), and *functional ability* (*n* = 1).

**Table 2:** Identified narrative categories with respective studies

Main categories	Sub-categories	Studies
***Psychosocial determinants***		
**Inadequate discharge planning**	PLWD living at home alone	Knox et al. (2021) [46], Callahan et al. (2012) [50], Tropea et al. (2017) [52]
	Insufficient post-discharge care resources	Knox et al. (2021) [46], Callahan et al. (2012) [50], Tropea et al. (2017) [52], Cummings et al. (1999) [45], Graversen et al. (2021) [16]
	Complex care	Cummings et al. (1999) [45]
**Interdisciplinary collaboration**	*Poor communication*: - Poor information sharing when transitioning healthcare facilities. - Short discharge timeframes	Gilmore-Bykovskyi et al. (2017) [55]
	*Good communication*: - Different healthcare professionals using their expertise to achieve the same goal of reducing hospital readmissions. - Primary care continuity between primary care professionals and PLWD and their carers.	Gustafsson et al. (2017) [56], Godard-Sebillotte et al. (2021) [17]
**Ethnic differences in dementia**	Socioeconomic disadvantages among PLWD of ethnic minorities	Gilmore-Bykovskyi et al. (2023) [57]
***Psychosocial and physical connection***		
**Behavioural and psychological symptoms**	Potential overuse to manage behavioural and psychological symptoms (BPSD) and delirium	Daiello et al. (2014) [53] Tannenbaum et al. [54]
	Hospitalised PLWD with behavioural symptoms	
***Physical determinants***		
**Functional ability**	Individual ability to mobilise and perform self-care tasks to reduce recurrent admissions	Knox et al. (2020) [47]
**Long-term conditions**	Common long-term conditions	Lin et al. (2017) [51], Sakata (2018) [18], Van de Vorst (2019) [19]
	Accumulating long-term conditions	Rudolph et al. (2010) [49]
	Common hospital admission aetiologies	Chang et al. (2015) [44]

### Psychosocial determinants of hospital readmissions in dementia

#### Inadequate hospital discharge planning

Five studies highlighted patterns of inadequate hospital discharge planning in older PLWD, where 30-day hospital readmission rates increased when older adults were discharged to live at home alone or after short-term domiciliary care [16, 45, 46, 50, 52]. The studies by Knox et al. [46] and Callahan et al. [50] identified that the primary reasons for unsuccessful discharges were due to unplanned hospital readmissions during and following domiciliary care, where PLWD were living at home alone. Tropea et al. [52] demonstrated similar results, where their cohort study identified increased 2, 7 and 28-day hospital readmission rates when people with dementia were discharged to their homes. This increase in hospital readmission rates resulted in a 47% increase in healthcare utilisation costs, compared to hospital readmissions among older people without cognitive impairment [52]. In a study where discharge plans developed by social workers were assessed for adequacy by primary care providers, Cummings et al. [45] identified factors that predicted hospital readmission for PLWD. Inadequate discharge plans included factors related to complex care (*p* < 0.01), problematic behaviours (*p* < 0.05), unrealistic family beliefs in the ability to provide care (*p* < 0.001), and unavailable resources (*p* < 0.01) [45]. In addition, research conducted by Graversen et al. [16] compared the risk of 30-day hospital readmission after a hospital discharge for pneumonia in PLWD and people without dementia. Graversen et al. [16] identified a 7% increase in hospital readmissions for PLWD compared to people without dementia, corresponding to an overall adjusted incidence rate ratio (aIRR) of 1.07 (95% CI 1.04–1.10). The highest hospital readmission rates were found within the first few days of hospital discharge for PLWD hospitalised with pneumonia [16]. The short time spent at home before hospital readmissions suggests inadequate hospital factors for PLWD, which include a lack of dementia-friendly hospital environments and poorly updated discharge planning [16].

#### Interdisciplinary collaboration

Three studies [17, 55, 56] highlighted contrasting examples of interdisciplinary collaboration between healthcare staff and older PLWD, which could act as a facilitator or barrier towards hospital readmissions in dementia. Gilmore-Bykovskyi et al. [55] interviewed nursing home staff about the needs of PLWD during hospital-to-nursing home transitions. Nursing home staff reported feeling ill-equipped with inadequate information sharing from hospital ward staff, short discharge timeframes, and limited control over nursing home admission discussions regarding the individuals’ care needs [55]. The nursing home staff acknowledged that hospital and nursing home staff may have different perceptions of the physical and psychosocial care needs for PLWD, such that these differences may contribute towards increased hospital readmissions [55].

As a corollary, a randomised controlled trial by Gustafsson et al. [56] examined whether clinical pharmacist participation in hospital ward rounds would reduce drug-related hospital readmission rates in PLWD. In the intervention group, the 30-day and 180-day hospital readmission rates were 5% and 11% compared to the control group readmission rates of 11% and 20%, when adjusted for patients with heart failure [56]. It was thought that such reduced drug-related hospital readmissions in PLWD were due to the close collaboration between the pharmacists and hospital ward medical team, where both areas of expertise worked together to reduce the occurrence of avoidable drug-related hospital readmissions [56].

Godard-Sebillotte et al. [17] also demonstrated adequate interdisciplinary collaboration as a key factor in reducing readmission, where they estimated the association between primary care continuity and avoidable hospital readmissions in PLWD. The results identified that when PLWD and their family carers attended regular primary care visits with the same physician, there was a reduced risk of 30-day hospital readmissions within the following year for PLWD (RR = 0.81; 95% CI 0.72–0.92; *p* < 0.001) [17].

#### Ethnic differences in dementia

An American cohort study by Gilmore-Bykovskyi et al. [57] examined the association between race and 30-day hospital readmissions among Medicare beneficiaries of Black and Non-Hispanic White individuals with dementia. The unadjusted analysis demonstrated that the odds of hospital readmission among Black beneficiaries were 37% greater compared to non-Hispanic White beneficiaries (OR 1.37, CI 1.35–1.39) [57]. Disability among Black beneficiaries was twice as high compared to the disability among non-Hispanic White beneficiaries, and a disproportionate number of Black beneficiaries lived in the most disadvantaged neighbourhoods across the USA (28.6% Black vs. 8.9% non-Hispanic White) [57]. After adjusting for all measured factors which included but were not limited to neighbourhood disadvantage, education level and long-term conditions, Black beneficiaries remained to have greater odds of hospital readmissions by 16% (OR 1.16, CI 1.14–1.17) [57]. Gilmore-Bykovskyi et al. [57] recognised the reduction of hospital readmission odds from 37% to 16% among Black beneficiaries, indicating that approximately 50% of the observed excess risk was attributable to exposures associated with racial differences that may include unmeasured clinical factors.

#### Behavioural and psychological symptoms

A cohort study conducted by Tannenbaum et al. [54] investigated the association of clinical outcomes with behavioural symptoms including wandering, agitation, aggression and psychosis in hospitalised PLWD. Within the cohort of hospitalised PLWD, 40.6% had behavioural symptoms [54]. Among this subgroup, 43.1% received a documented diagnosis of delirium, and 30.9% were prescribed antipsychotic medication [54]. Notably, only 0.2% of PLWD with behavioural symptoms had a formal diagnosis of ‘*dementia with behavioural symptoms’* recorded in their medical notes. Tannenbaum et al. [54] observed that hospitalised PLWD exhibiting behavioural symptoms were more likely to be admitted from a care facility (26.6% vs. 23.7%, *p* < 0.05). This specific cohort of patients demonstrated a higher tendency for readmission to acute medical services, compared to surgical services (92.7% vs. 91%, *p* = 0.003). Notably, hospital factors with this group included the presence of an indwelling catheter (11.1% vs. 6%, *p* < 0.001), an elevated falls risk (46.7% vs. 42.3%, *p* < 0.002), bed alarms attached to their hospital beds (81.6% vs 77.4%, *p* < 0.001), and Do Not Resuscitate (DNR) orders (40.6% vs 33.1%, *p* < 0.001) [54]. As a result, this study concluded that hospitalised PLWD with behavioural symptoms exhibited a moderate increase in their odds of having 30-day hospital readmissions (OR = 1.14, CI 95% 1.014–1.289) [54].

The frequent use of antipsychotic medication for behavioural and psychological symptoms in older PLWD may act as a determinant of hospital readmissions, which was identified in the cohort study by Daiello et al. [53]. Daiello et al. [53] investigated the association of dementia with early hospital readmissions among a cohort of Medicare health insurance beneficiaries. This study highlighted that PLWD with 30-day hospital readmissions had more frequent prescriptions for antipsychotic medication, having at least two prescriptions within the study period of one year (12.7% readmission vs. 9.2% no readmission *p* < 0.001) [53]. Additionally, frequent use of antipsychotic medication in the 6 months before or after the index hospitalisation was associated with higher odds of 30-day hospital readmissions for PLWD [53]. Although not investigated in the study, Daiello and Colleagues [53] acknowledged that recurrent use of antipsychotic medication in PLWD may be a marker of unmeasured psychosocial factors including behavioural symptoms, agitation and delirium, which increases the risk of 30-day hospital readmissions. Nevertheless, this acknowledgement is supported by the results of Tannenbaum et al’s study [54].

### Physical determinants of hospital readmissions in dementia

#### Functional ability

A cohort study by Knox et al. [47] outlined functional ability in PLWD as a determinant for hospital readmissions, as they examined the association between mobility, self-care and caregiver support with 30-day hospital readmissions in PLWD. The study revealed that PLWD with domiciliary care who were most dependent on mobility (OR 1.59, 95% CI 1.47–1.71) and self-care (OR 1.73, 95% CI 1.61–1.87), had the highest odds for 30-day hospital readmissions when adjusted for caregiver support [47]. In the most dependent quartiles for mobility and self-care, the two most common conditions resulting in hospital readmissions were sepsis and urinary tract infections [47]. This finding suggests that regardless of the level of caregiver support and dementia severity, the individuals’ ability to function in the form of mobility and self-care, determines their risk of 30-day hospital readmissions.

#### Long-term conditions

Long-term conditions that affect PLWD were commonly listed as reasons for hospital readmissions in five studies [18, 19, 44, 49, 51]. The long-term conditions included cardiovascular disease (CVD), pneumonia, urinary tract infections and falls [18, 19, 44, 49, 51]. The retrospective cohort studies by Lin et al. [51] and Sakata et al. [18] retrieved similar results, where over half of the most common hospital admission reasons which included various long-term conditions, increased the hospital readmission rate for PLWD. Lin et al. [51] highlighted a 73% all-cause 30-day readmission rate, where PLWD were readmitted to hospital with different comorbidity problems from their index admission. When investigating the impact of CVD on hospital readmissions, Van de Vorst et al. [19] recognised that 37% of hospitalised PLWD and 50% of day clinic outpatients with dementia both with CVD, experienced hospital readmissions within one year. A longitudinal study by Rudolph et al. [49] demonstrated that the cumulative risk for rehospitalisation increased with the number of long-term conditions that older PLWD live with [49]. The authors reported that the risk of rehospitalisation was 37% with no long-term conditions, 57% with one long-term condition, 70% with two or three long-term conditions, and 80% with four or five long-term conditions [49]. However, when Chang et al. [44] explored the roles of systemic diseases and hospital admission aetiologies for predicting hospital readmissions in dementia, they outlined contrasting results. Hospital admission aetiologies were found to have more clinical weighting than co-existing medical conditions when predicting hospital readmission in older PLWD [44]. Nevertheless, a significantly smaller sample of 203 PLWD participated in this study, which may reflect the difference in comorbidity-related hospital readmissions in the previously mentioned studies, which included larger samples of 50,000 to 2 million PLWD [18, 19, 49, 51].

## Discussion

This narrative review provided an overview of some of the psychosocial determinants that contribute towards hospital readmissions in older PLWD, in addition to the known physical determinants. Over half of the publications in this review highlighted psychosocial determinants that are likely to increase the risk of hospital readmissions in dementia [11, 17, 45, 16, 46, 50, 52, 55–57]. These psychosocial determinants included inadequate hospital discharge planning particularly when PLWD were discharged to their homes [46, 50, 52], insufficient information sharing of care needs between healthcare staff in hospital and community settings [55], and socioeconomic disadvantages among certain ethnic minority groups [57]. Six of the 17 articles in this review outlined the physical determinants that are known to increase the risk of hospital readmission for PLWD, which included living with common long-term conditions such as cardiovascular disease and living with an accumulation of long-term conditions throughout the dementia diagnosis [18, 19, 44, 47, 49, 51]. Consequently, reduced mobility and the functional ability to perform everyday activities such as self-care tasks, are also known to increase hospital readmissions in older PLWD [47]. While this review highlighted the elevated risk of hospital readmissions associated with the coexistence of multiple physical conditions, reduced mobility and function, the contribution of frailty in older individuals with dementia was overlooked in the included studies. However, frailty in PLWD is associated with an increased risk of hospital readmissions, as demonstrated by an observational study conducted by Briggs et al. [58]. Some articles that highlighted physical determinants of hospital readmissions acknowledged the importance of psychosocial determinants that may increase the risk of rehospitalisation in older PLWD [18, 47, 51, 53]. The authors discussed that where common acute conditions may increase the risk of hospital readmissions, these readmissions could also be indicative of problems in the continuity of care, inefficient resource use, and the unrecognised need for special discharge planning in acute care [18, 47, 51, 53].

### Psychosocial and physical connection of hospital readmissions in dementia

The frequent use of antipsychotic medication in older PLWD was recognised to explain the interplay between the psychosocial and physical determinants of hospital readmissions [53]. Antipsychotic medication is primarily used for psychosis in mental health conditions including schizophrenia and bipolar disorder [59]. However, antipsychotic medication was additionally licensed in the 1950s for short-term use to manage behavioural and psychological symptoms of dementia (BPSD), following ineffective non-pharmacological treatments [59–61]. Our review raises concerns about the frequent use of antipsychotic medication in older PLWD, as they are sensitive to the extrapyramidal side effects which include Parkinson-like tremors, involuntary muscle contractions and QT prolongation (interference with conduction in the heart) [59, 60]. These side effects of antipsychotic medication in dementia increase the risk of other common side effects including falls, sedation, and cognitive decline, which may increase the length of hospital stay and consequently accumulate additional care needs [61]. Daiello et al. [53] demonstrated the consequences of the side effects that follow repeated use of antipsychotic medication, as older PLWD with 30-day hospital readmissions were more likely to have at least two prescriptions for antipsychotic medication within one year. Even though an association between psychosocial and physical determinants of hospital readmissions in dementia exists with a plausible underlying theory, there is still limited evidence to support this statement.

### Fragmented health and social care

This review highlights fragmented care between health and social care professionals leading to poor distribution of care responsibilities, which results in increased rates of hospital readmission for older PLWD [17, 18, 55]. The *Global Action Plan for Dementia* by the World Health Organization emphasises the need for all countries to shift the focus of care from hospitals towards community-based settings, that integrate health and social care systems to provide evidence-based care for PLWD [62]. This pledge was reiterated in the *Prime Minister’s Challenge on Dementia 2020*, where the UK Government committed to improving health and social care provision in the community to reduce avoidable hospital readmissions in PLWD [63]. In the UK, access to NHS healthcare is free, whereas access to social care is means-tested and limited [64]. This can result in certain populations having difficulty in accessing adequately funded social care, including older PLWD [64]. Sir Marmot’s review titled *Health Inequity in England: The Marmot Review 10 Years On*, found increasingly deprived areas in the North, Midlands and Southern coastal towns of England [65]. Older PLWD living in these socioeconomically deprived areas do not live in environments that support social connectedness, physical health and activity, or mental stimulation [64, 65].

For many individuals living with dementia, adjusting to the evolving circumstances during the Covid-19 pandemic involved limited or no access to in-person community healthcare and social activities. A qualitative study conducted by Giebel et al. [66] showed that multiple lockdowns and temporary closures of routine care resulted in a decline in mental stimulation, physical deterioration and heightened dependency among PLWD. Moreover, family carers experienced a decline in both mental and physical health, as they were compelled to provide caregiving responsibilities that would have typically been handled by paid carers [66].

However, the wider health and social care ramifications of Covid-19 are currently being realised. Since in-person healthcare services reopened, PLWD were found to have more advanced dementia with additional physical and psychological care needs, and increased risk of further hospital admissions [67, 68]. Digital healthcare was widely implemented to mitigate hospital readmissions such as using smart home devices and telemedicine. Determining the types of technology that are both suitable and safe for PLWD requires further investigation [69, 70].

### Strengths and limitations

A major strength of this review is that to our knowledge, this is the first narrative review to focus on the psychosocial determinants of hospital readmissions in PLWD. Although hospital readmissions in PLWD have been previously addressed in research, the physical determinants have gained more attention than other determinants of hospital readmissions [11, 18, 53, 71]. Our review demonstrated the importance of some of the psychosocial impacts of dementia in addition to the known physical impact.

This review also adopted the methodological framework devised by Arksey and O’Malley [26], to guide a systematic approach and promote scientific rigour. Arksey and O’Malley were the first scholars to develop an internationally recognised framework that follows an iterative process, to clarify the usefulness and methodology of reviews of an exploratory nature. This review also utilised the Preferred Reporting Items for Systematic Reviews and Meta-Analyses extension for Scoping Reviews (PRISMA-ScR) [27], to provide a guided report of the narrative review.

Limitations of this review include that the majority of publications were conducted within Western society. Only two studies in this review were from Japan and Taiwan [18, 44], compared to eight studies from the USA [45–47, 49–51, 53, 55]. As healthcare systems operate differently around the world, future research should be conducted for non-Western societies with rapidly ageing populations living with dementia. Secondly, it is important to note that the data collected in this review span the period from 1991 to 2019, despite studies being published up to 2023. Hence, such data may not provide a comprehensive reflection of the current understanding of hospital readmissions in PLWD, considering that the data were collected before the onset of the Covid-19 pandemic. Lastly, there was heterogeneity within the studies including different study samples, study aims and outcome measures. Hence, a meta-analysis was not conducted in this review.

### Future recommendations for research

Future recommendations for research should highlight the voices of healthcare staff and family carers of older PLWD, within different care settings. This review only managed to obtain the experiences and perceptions of care transitions for PLWD from nursing home staff [55]. Although the interviews highlighted problems resulting in 30-day hospital readmissions for older PLWD, there was a lack of varied voices representing dementia care. Many of the publications in this review discuss the barriers of access to care, limited care resources within hospital and community settings, and problems with hospital discharge planning [16, 18, 46, 51, 55]. However, little is known about why these problems exist. Therefore, future discussions with healthcare staff and family carers involved in dementia care could highlight the underlying issues of hospital readmissions among older PLWD.

Future research should focus on providing evidence to explain the interactions between the psychosocial and physical determinants of hospital readmissions in older PLWD, to address the root cause of this problem. Despite attempting to identify and investigate emerging links between psychosocial and physical determinants, the literature contained a dearth of data to allow us to draw conclusions. One would assume that significant synergistic or at least additive interactions exist between physical and psychosocial factors, perpetuating high rates of hospital readmissions for PLWD. However, this will need to await studies to be specifically designed to answer such important questions. Only by understanding such potential interactions would one be able to deliver a holistic intervention approach to decrease rates of hospital readmissions for PLWD. Unfortunately at this time, there is limited evidence to demonstrate how managing both the psychosocial and physical determinants of dementia can help reduce hospital readmissions in older PLWD. This is to recognise that health outcomes according to each determinant of health are nuanced and complex [72]. Nevertheless, effective interventions to help manage these determinants together could support the multi-factorial problem of hospital readmissions for older PLWD. Future research should highlight appropriate approaches to help tackle this problem.

Despite our systematic approach to the literature search, only few psychosocial factors were identified. One can assume that several other psychosocial factors must exist that could have an impact on hospital readmission rates for PLWD. The list of such factors includes but is not limited to loneliness, poverty, education, mental wellbeing, willingness to accept help and family support. Many psychosocial factors have been suspected as potentially being implicated in hospital readmissions in PLWD, which include being an immigrant, male sex, having behavioural symptoms and frequent use of antipsychotic medication [11]. Interpersonal factors of dementia care were also highlighted, as living with fewer cohabitants, problems with discharge care transitions, high caregiver stress and inadequate caregiver support were reported to contribute to increased hospital readmissions. Future research needs to assess these and other psychosocial factors individually, in combination with each other as well as the other implicated physical factors.

## Conclusion

This review provides insight into how few psychosocial determinants may result in increased hospital readmissions for PLWD. The determinants include inadequate integrated care and insufficient care planning between care settings and healthcare professionals. Future research should try to identify the impact of a myriad of other psychosocial factors and focus on the interaction between the psychosocial and physical determinants, and multidisciplinary interventions across care settings to reduce acute recurrent admissions in dementia.

### Supplementary Information

**Supplementary Material 1.**

## Data Availability

All data generated or analysed during this study are included in this published article and its supplementary information files.
